# Tumor-Suppressive microRNA Therapy Inhibits Growth of Glioblastoma Multiforme Xenografts

**DOI:** 10.3390/cancers18091479

**Published:** 2026-05-04

**Authors:** Ezgi Biltekin, Sayra Dilmac, Nermin Kahraman, Ogun Ali Gul, Yasemin M. Akay, Zhihui Wang, Metin Akay, Bulent Ozpolat

**Affiliations:** 1 Department of Nanomedicine, Houston Methodist Research Institute, Houston, TX 77030, USA; ebilteki@central.uh.edu (E.B.);; 2 Department of Biomedical Engineering, University of Houston, Houston, TX 77204, USA; 3 Stephenson School of Biomedical Engineering, The University of Oklahoma, Norman, OK 73019, USA; 4 Mathematics in Medicine Program, Houston Methodist Research Institute, Houston, TX 77030, USA; 5 Department of Experimental Therapeutics, The University of Texas MD Anderson Cancer Center, Houston, TX 77030, USA

**Keywords:** glioblastoma multiforme, GBM, microRNA, miRNA, miRNA therapeutics, eEF2K, FOXM1, AXL, gene regulation

## Abstract

Glioblastoma multiforme (GBM) is defined by rapid progression, high invasiveness, and a poor prognosis, with a median survival of only ≅13 months despite current treatments. Its marked genetic heterogeneity, high mutational burden, and cancer stem cell population make GBM exceptionally difficult to treat, highlighting the urgent need for more effective, multitargeted therapies. Non-coding RNAs, particularly tumor suppressor microRNAs (miRNAs), have gained attention for suppressing key oncogenic processes that drive tumorigenesis, metastasis, and drug resistance, positioning them as promising tools for targeting multiple oncogenic pathways. We recently found that FOXM1/AXL-eEF2K collaboratively drive GBM cell proliferation, survival, and invasion through the formation of a signaling hub complex. In this study, we employed miRNA prediction algorithms to identify a specific miRNA, in vitro functional assays and in vivo GBM flank model to target GBM tumorigenesis by distrupting the FOXM1/AXL-eEF2K signaling hub. Our results indicated that FOXM1, AXL, and eEF2K are overexpressed in GBM patient tumors. To target the FOXM1/AXL-eEF2K signaling hub, we identified miR-449b-5p, miR-329-3p, and miR-518c as potential co-inhibitors of FOXM1/AXL-eEF2K and suppressors of cell proliferation, migration–invasion, and spheroid formation. Furthermore, the combination of miR-449b-5p, miR-329-3p, and miR-518c treatments with temozolomide led to synergistic enhancements in cell proliferation suppression and the induction of apoptosis and ferroptosis. More importantly, in vivo miR-329-3p treatment led to remarkable suppression of GBM tumor xenografts. These findings indicate that miR-329-3p-based tumor suppressor therapy may offer a multitargeted approach for GBM treatment.

## Simple Summary

Glioblastoma multiforme (GBM) is the most aggressive type of glioma and is characterized by its highly invasive nature, therapy resistance and the lack of effective targeted therapies, with an average life expectancy of ≅13 months. microRNAs (miRNAs) are small non-coding RNAs that are able to regulate their target mRNAs by specifically binding to their 3′ UTRs and are attracting increasing interest as a promising strategy for targeting key oncogenic processes. We recently demonstrated that the FOXM1/AXL-eEF2K signaling axis is an important regulator of GBM cell proliferation, motility, stemness and cell death. In this study, we investigated multitargeting strategies by identifying miR-449b-5p, miR-329-3p, and miR-518c as potential co-inhibitors of the FOXM1/AXL-eEF2K axis and regulators of cell proliferation, motility, stemness and cell death. Moreover, combinatory treatment of miR-449b-5p, miR-329-3p, and miR-518c with temozolomide (TMZ), the standard chemotherapeutic agent, synergistically enhanced the effect of TMZ. The in vivo administration of miR-329-3p significantly suppressed tumor growth in GBM tumors. Although our study is limited to a flank xenograft model, this study highlights miR-based multitarget strategies as a promising approach for GBM.

## 1. Introduction

Glioblastoma multiforme (GBM) is an IDH wildtype CHS WHO Grade 4 glioma and is recognized as a mostly incurable, highly aggressive primary brain tumor that is associated with rapid progression, high invasiveness, and poor prognosis [1,2]. GBM is the most common brain malignancy, and about 50% of primary malignant brain tumor patients are diagnosed with GBM. Unfortunately, the median survival is 13 months after diagnosis [3,4,5]. Despite current treatment strategies, including surgery, radiotherapy (RT), and chemotherapy, temozolomide (TMZ) remains the first-line chemotherapeutic agent for glioblastoma. However, even in combination with other therapeutic regimens, the 5-year survival rate remains dismally low, at approximately 7% [3,5,6,7,8,9,10]. As a result, GBM remains highly challenging to treat, underscoring the urgent need for the development of multitargeted therapies to improve the dismal patient survival rate. The major reasons for treatment failures in GBM patients include the significant genetic heterogeneity, the high number of mutations, and the lack of molecular targets and effective targeted therapeutics. Moreover, the protective blood–brain barrier limits drug delivery, and drug resistance mechanisms due to enhanced DNA repair, activation of alternative signaling pathways, the abundance of the cancer stem cell population, and the protective tumor microenvironment represent challenges to current treatment strategies [11,12,13].

MicroRNAs, a subgroup of small non-coding RNAs that are around 22 nucleotides in length, have been widely studied for their gene regulatory role in cancers and have been shown to be capable of regulating multiple mRNAs by binding specific binding sites at the 3-untranslated region (UTR) [14]. In recent years, non-coding RNAs have been recognized for their hallmark roles in cancer tumorigenesis, progression, and drug resistance [15,16,17,18]. In GBM and other human cancers, miRNAs play crucial roles by acting as both oncogenes (oncomiRs) and tumor suppressors, regulating the expression of tumor suppressor or oncogenes, respectively, and thus contribute to tumor progression and treatment response.

Eukaryotic Elongation Factor 2 Kinase (eEF2K) and AXL-tyrosine kinase signaling are emerging oncogenic signaling pathways that play a role in cell proliferation, metastasis, and therapy resistance in solid cancers [19,20,21,22]. The Forkhead box transcription factor FOXM1 promotes stemness and radiotherapy resistance [23] and is another potential therapeutic target in GBM. We recently showed that the FOXM1-AXL/eEF2K signaling axis regulates cell proliferation, motility and spheroid formation capabilities of GBM through regulation of each other by the orchestration of FOXM1. Furthermore, inhibition of the FOXM1-AXL/eEF2K axis promoted apoptosis and ferroptosis alone or in combination with TMZ in GBM cells. These results underlined their potential for multitargeting strategies, and in vivo inhibition of the FOXM1-AXL/eEF2K axis has been proposed as a potential therapeutic strategy in GBM [24,25]. Importantly, there are currently no FDA-approved inhibitors for targeting these oncogenic signaling molecules.

In this study, we showed that FOXM1, AXL, and eEF2K are highly expressed in GBM patient tumors, and through an extensive search of miRNA and mRNA interaction algorithms and databases, we identified several miRNAs, including miR-449b-5p, miR-329-3p, and miR-518c, that directly target FOXM1, eEF2K, and AXL mRNAs at the 3′-UTRs. Our functional assays demonstrated that these miRNAs inhibited cell proliferation, spheroid formation, and cell migration/invasion and induced apoptosis. We also demonstrated that miR-449b-5p, miR-329-3p, and miR-518c, through targeting eEF2K, AXL, and FOXM1, synergistically enhanced the efficacy of TMZ and caused a significant induction of apoptosis and ferroptosis. More importantly, in vivo therapeutic administration of miR-329-3p effectively suppressed tumor growth of xenografts in mouse models. These findings highlight the potential of miRNA-based, multitargeted therapeutic approaches and support their integration with standard chemotherapy regimens in GBM management.

## 2. Materials and Methods

### 2.1. Cell Lines and Cell Culture

The human glioblastoma multiforme cell lines U87, LN229, U373, and U118 were obtained from ATCC. The U87, LN229, U373, and U118 cell lines were cultured in Dulbecco Modified Eagle medium (DMEM)/F12 medium (Corning, Tewksbury, MA, USA) supplemented with 10% fetal bovine serum (FBS) and 1% penicillin–streptomycin solution (Sigma, St. Louis, MO, USA). Cells below passage 10 were used for experiments and were incubated at 37 °C in a 95% air and 5% CO_2_ saturated atmosphere and tested regularly to prevent mycoplasma contamination.

### 2.2. Colony Formation Assay

U87, LN229, U373, and U118 cells were prepared as single-cell suspensions and seeded at a density of 1 × 10^3^ cells/ml in 12-well plates. Forty-eight hours later, the media were removed, and the attached cells were treated once with 25 nM miRNA (miR-449b-5p, miR-329-3p, miR-518c) and cultured for 10–14 days. After colony formation, colonies were stained with 10% crystal violet and quantified by using ImageJ (Version 1.54g).

### 2.3. Transfection with miRNA

miR-449b-5p (ID: MC11521), miR-329-3p (MC10453), and miR-518c (MC10882) were purchased from Thermo Fischer Scientific (Waltham, MA, USA). Specific control non-silencing siRNA and control mimic miRNA were used as non-inhibitory scrambled RNA controls. U87-MG and LN229 cells were seeded at a density of 1–1.25 × 10^5^/well in 6-well plates. Twenty-four hours later, the cells treated with 100 nM miRNA in DMEM/F12 medium in the absence of FBS with the facilitation of the HiPerFect transfection reagent (Qiagen, Germantown, MD, USA). A 4–6 h incubation period was completed with the addition of 10% FBS to each well, and the cells were incubated for another 72–96 h.

### 2.4. Protein Extraction and Western Blot Analysis

U87, LN229, U373, and U118 cells were harvested 72 h after transfection with miRNAs. Cell pellets were processed and lysed with RIPA buffer supplemented with 1% phosphatase and 1% protease inhibitor cocktails as described earlier [25]. For Western blot analysis, equal amounts of protein (40 ug) were resolved by SDS-PAGE, transferred to membranes overnight, and blocked with 5% non-fat dry milk in Tris-buffered saline containing 0.1% Tween-20 (TBS-T) for one hour at room temperature. Membranes were incubated overnight at 4 °C with primary antibodies against eEF2K, GPX4 and GAPDH (Cell Signaling Technology, Danvers, MA, USA), AXL (R&D Systems, Minneapolis, MN, USA), and FOXM1 (Santa Cruz Biotechnology, Dallas, TX, USA). The membranes were washed and then incubated for 1 h at room temperature with horseradish peroxidase-conjugated secondary antibodies (anti-rabbit or anti-mouse, Cell Signaling Technology). Protein bands were visualized using the ChemiDoc™ Imaging System (Bio-Rad, Hercules, CA, USA) in combination with the Immobilon^®^ Classico Western HRP Substrate (Millipore Sigma, Burlington, MA, USA).

### 2.5. Tumorosphere Formation Assay

U87 and LN229 cells were treated with 100 nM miRNA (control, miR-449b-5p, miR-329-3p, or miR-518c) for 72 h. After the cells were collected, single-cell suspensions in complete MammoCult Medium (StemCell Technologies, Vancouver, BC, Canada) were prepared and seeded in ultra-low attached 6-well plates at a density of 1 × 10^4^/well in 2 mL as duplicates. The evolution of the spheroids was captured from three different random representative areas by an inverted microscope (EVOS^®^ FL) at 4× objective every 24 h. At 120 h, spheroids with a diameter >70 μM in each well were counted to measure the number of spheroids.

### 2.6. Cell Migration-Scratch Assay

The wound healing/cell scratch assay was used for measuring cell motility and migration. U87 and LN229 cells were seeded at a density of 1 × 10^−5^/well in 6-well plates. Twenty-four hours later, miRNA (control miR, miR-449b-5p, miR-329-3p or mir-518c) transfections were performed as described. Seventy-two hours after treatment, a scratch was created with a sterile 200 uL pipette tip to the monolayer of treated cells. Then, each well was gently washed with medium to remove detached cells, and fresh medium was added. Cells in the scratched area were observed, and images were taken at the 0 h, 24 h, and 36 h. with an EVOS^TM^ FL microscope. The open area between the two sides of a scratch was measured with ImageJ. The results were calculated as a percentage of the closed area.

### 2.7. In Vitro Matrigel Invasion Assay

U87 and LN229 cells were transfected with 100 nM miRNA (miR-449b-5p, miR-329-3p, or mir-518c). Seventy-two hours after treatment, the cells were collected, and 5 × 10^5^ live U87 cells per chamber and 8 × 10^5^ live LN229 cells per chamber were seeded in serum-free medium in each matrigel-coated Matrigel Invasion Chamber (BD Biosciences, San Jose, CA, USA). Cells were allowed to invade for 48 h through the 10% FBS-supplemented medium in the lower part of the chamber. Then, the inserts were fixed with Hema3 Fixative (FisherBrand, Pittsburg, PA, USA). The number of invaded cells was counted using a light microscope from 4 different fields.

### 2.8. Apoptosis Detection with Annexin V Assay

The Annexin V assay was used for the determination of apoptosis. U87-MG and LN229 cells were seeded in 6-well plates and transfected with 100 nM miRNA (control miR, miR-449b-5p, miR-329-3p, or mir-518c). Ninety-six hours after treatment, the cells were collected with the medium they had been incubated in and then were analyzed by annexin V/propidium iodide staining according to the manufacturer’s protocol (FITC–Annexin V kit; BD Pharmingen, San Diego, CA, USA). FITC-labeled cells were analyzed by flow cytometry at the Houston Methodist Research Institute, Flow Cytometry Core.

### 2.9. Lipid Peroxidation Assay

To detect ferroptosis, a lipid peroxidation assay was used with the help of BODIPY™ 581/591 C11 (Lipid Peroxidation Sensor) (Invitrogen™, Carlsbad, CA, USA) staining following the manufacturer’s protocol. LN229 and U87 cells were seeded in 6-well plates and were treated 24 h later with control miR, miR-449b-5p, miR-329-3p or mir-518c (100 nM) for 96 h. For TMZ and miR combination assays, following 24 h of miR treatment, TMZ (150 µM or 200 µM) was added, and the total treatment time of 96 h was completed. At the end of the experiment, the cells were collected and stained with BODIPY/DAPI and analyzed by flow cytometry at the Houston Methodist Research Institute, Flow Cytometry Core.

### 2.10. Temozolomide and miR Combination Therapy and Synergistic Effect Assessment

The LN229 and U87 cell lines were seeded in 96-well plates (1250 cells/well). Twenty-four hours later, the cells were transfected with miRNA (miR-449b-5p, miR-329-3p or mir-518c) at concentrations of 100 nM, 50 nM, 25 nM, 12.5 nM and 6.25 nM. Forty-eight hours after miR transfection, Temozolomide was added using the serial dilution method from 400 uM to 50 uM and 300 uM to 35 uM. Forty-eight hours after temozolomide treatment, cell viability was measured by the MTS [3-(4,5-dimethylthiazol-2-yl)-5-(3-carboxymethoxyphenyl)-2-(4-sulfophenyl)-2H-tetrazolium; Promega, G111] (Madison, WI, USA) and PMS (phenazine methosulfate) (Sigma-Aldrich, St. Louis, MO, USA) assay according to the manufacturer’s protocol, and the absorbance was read at 490 nm on an ELISA plate reader. The results were assessed with the online tool SynergyFinder Plus to determine the synergistic effect between miR treatment and Temozolomide as described [26].

### 2.11. In Vivo Xenograft GBM Tumor Model

For the in vivo GBM tumor model, ethical approval was obtained from the Houston Methodist Research Institute Animal Care and Use Committee (IACUC). Nude athymic female mice (4–5 weeks old) were purchased from Taconic Biosciences, and 1.5 × 10^6^ LN229 cells and 2.25 × 10^6^ U87 cells in 20% Matrigel were injected into the right flank of the nude mice. In the second week after GBM cell administration, when tumor sizes measured between 30 and 50 mm^3^, control miR, miR-449b-5p and miR-329-3p were incorporated into liposomes composed of 1,2-dimyristoyl-sn-glycero-3-phosphocholine (DMPC) and pegylated distearoyl-phosphatidylethanolamine (DSPE-PEG-2000; Avanti Lipids) at a dose of 0.15 mg/kg equivalent. Treatment twice a week was started. Single lipid nanoparticles were prepared as described earlier [27]. For each treatment, 8 uL/mouse miRNAs conjugated with lipid nanoparticles was administrated every week, and tumor progression was measured twice a week. After the completion of treatment, the mice were euthanized, and tumor tissues were collected and photographed. Tumor tissues were used for Western blot analysis for further investigation of the molecular mechanism.

### 2.12. TUNEL Assay

DNA fragmentation detected in apoptotic cells was measured by the TUNEL assay according to the manufacturer’s instructions. Tumor tissue sections were incubated with biotin-dUTP and terminal deoxynucleotidyl to detect DNA fragmentation. Positively stained cells were examined using an inverted microscope, and the number of TUNEL-positive cells in each field per section was quantified.

### 2.13. Immunohistochemistry (IHC)

Tumor samples that were resected from the mice were fixed in formalin, embedded in paraffin, and sectioned and stained with hematoxylin and eosin or Ki-67 to evaluate intratumoral cell proliferation, according to the manufacturer’s protocol. The slides were analyzed by microscopy (Zeiss, Carl Zeiss, Oberkochen, Germany).

### 2.14. Statistical Analysis

Data are presented as the mean ± standard deviation using at least duplicate/triplicate samples. Statistical significance was assessed using one-way analysis of variance (ANOVA) or *t*-test, with *p*-values less than 0.05 considered statistically significant. All statistical analyses were performed using GraphPad Prism version 9.5.1.

## 3. Results

### 3.1. FOXM1, AXL, and eEF2K Are Upregulated in GBM Patient Tumors 

**Figure 1:**
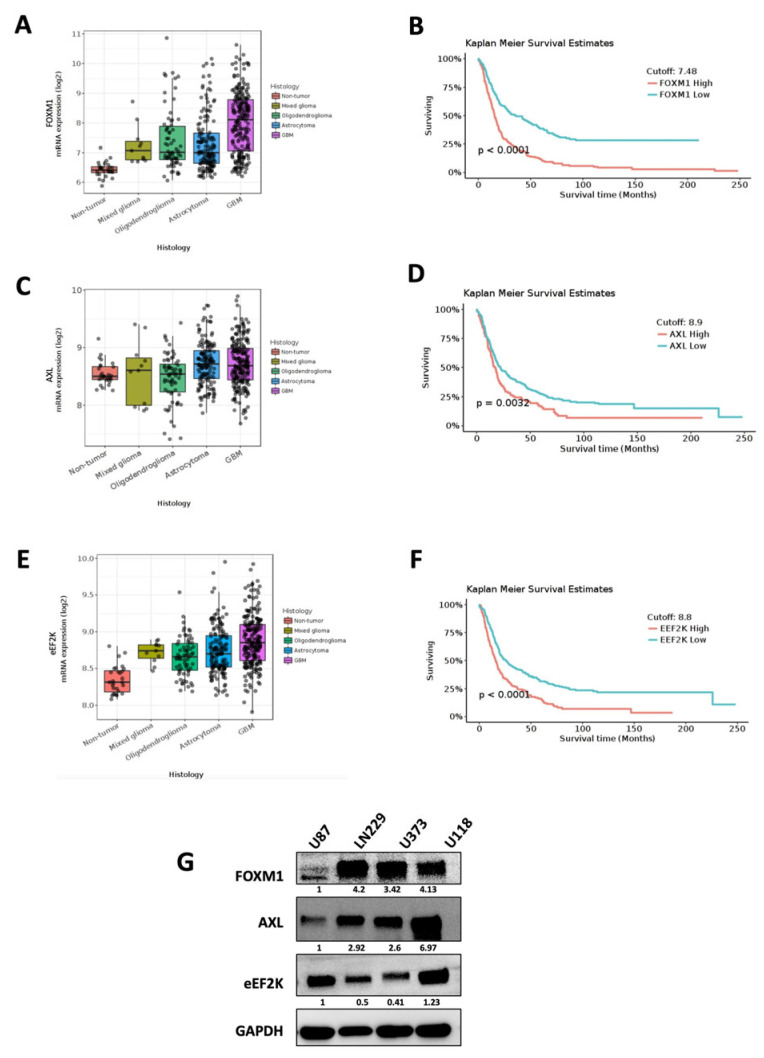
FOXM1, eEF2K, and AXL expression in GBM patient tumors and patient survival in combined brain cohort. (**A**–**F**) The Rembrandt brain tumor patient dataset was evaluated and indicated that FOXM1 (**A**), AXL (**C**), and eEF2K (**E**) are overexpressed in GBM tumor samples compared to non-tumor, mixed glioma, oligodendroglioma and astrocytoma tumor samples. Data are presented as mRNA expression (log2). Kaplan–Meier survival analysis indicated that higher FOXM1 (**B**), eEF2K (**D**), and AXL (**F**) expression levels in combined brain tumor cohort are associated with shorter overall patient survival (*p* < 0.0001, *p* = 0.0032, *p* < 0.0001). (**G**) Western blot analysis of GBM cell lines (LN229, U87, U373, U118) showed different levels of FOXM1, eEF2K, and AXL expression. Original western blots are presented in Figure S3.

To assess the clinical significance of FOXM1, AXL and eEF2K, we evaluated their mRNA expression in a publicly available brain tumor database (REMBRANT and CCGA Patient Dataset) (https://gliovis.bioinfo.cnio.es/) and performed Kaplan–Meier survival analysis. Our analysis revealed that FOXM1, AXL and eEF2K are highly overexpressed in GBM patient tissue samples compared with non-tumor, mixed glioma, oligodendroglioma and astrocytoma tumor tissue samples (Figure 1A,C,E) (Supplementary Figure S1). Kaplan–Meier survival analysis revealed that higher expression of FOXM1, AXL, and eEF2K in the combined brain tumor cohort was associated with shorter patient survival and poorer prognosis (*p* < 0.0001, *p* = 0.0032, *p* < 0.0001; Figure 1B,D,F). However, this association did not reach statistical significance when each tumor subtype was analyzed separately. We also evaluated the expression of FOXM1, AXL and eEF2K in GBM cell lines (LN229, U87, U373 and U118) and found different expression levels in different GBM cell lines, representing the inter-tumoral heterogeneity of GBM patients (Figure 1G).

### 3.2. Identification of miRNAs with Predicted Specific Binding to the 3’-UTRs of eEF2K, AXL and FOXM1 mRNAs

**Figure 2:**
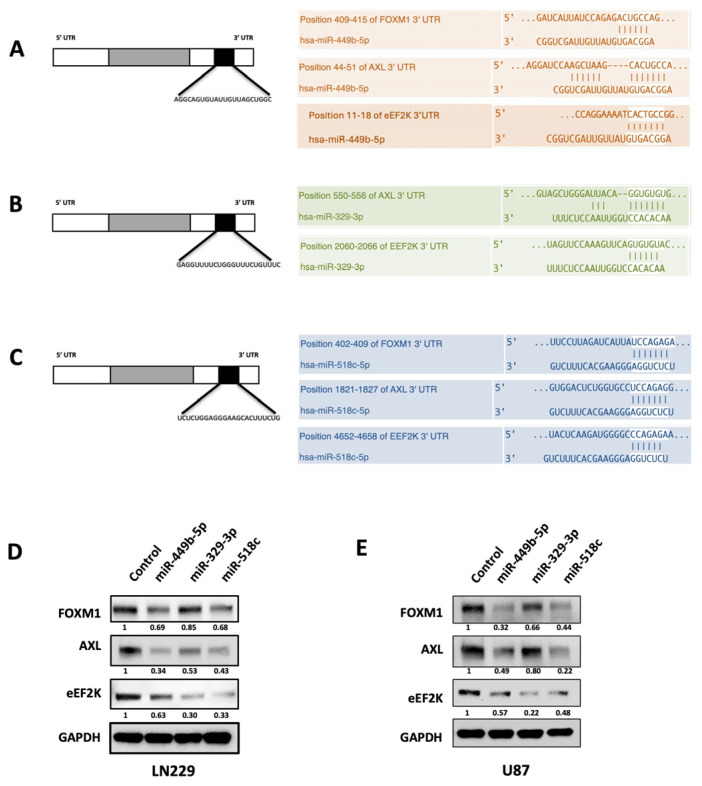
miR-449b-5p, miR-329-3p and miR-518c target prediction and the effect of miRNA treatment on FOXM1, eEF2K, and AXL expression in GBM cells. miRNA target prediction algorithms such as Target Scan, Diana Tools and miRDB were used to identify unique miRNAs that co-target FOXM1, eEF2K, and AXL mRNAs. (**A**) miR-449b-5p predicted to target specific mRNA binding consensus sequences at 3′-UTR of FOXM1, eEF2K, and AXL mRNAs. (**B**) miR-329-3p was identified as directly co-targeting AXL and eEF2K mRNA by a specific binding motif at 3′-UTR. (**C**) miR-518c was identified for its binding site targeting FOXM1, AXL and eEF2K mRNA. (**D**,**E**) Western blot analysis revealed that miR-449b-5p, miR-329-3p and miR-518c suppress the expression of FOXM1, AXL and eEF2K in LN229 and U87 GBM cell lines. Original western blots are presented in Figure S3.

To identify potential miRNA candidates for co-targeting FOXM1, AXL, and eEF2K, we utilized microRNA target prediction algorithm tools, including miRDB, DIANA TOOLS, and Target Scan and found that miR-449b-5p, miR-329-3p, and miR-518c have specific binding sites in the 3’-UTR of eEF2K, AXL, and FOXM1 mRNA and demonstrate the potential to co-target FOXM1, AXL, and eEF2K. miR-449b-5p has a predicted binding site in the 3′-UTRs of FOXM1, AXL, and eEF2K mRNAs (Figure 2A), while miR-329-3p is predicted to bind to the 3′-UTRs of AXL and eEF2K (Figure 2B), and miR-518c is predicted to bind to the 3′-UTRs of FOXM1, AXL, and eEF2K, as shown in Figure 2C [28,29,30,31,32].

### 3.3. miR-449b-5p, miR-329-3p, and miR-518c Suppress Expression of eEF2K, AXL and FOXM1 in GBM Cell Lines

To elucidate the regulatory role of miR-449b-5p, miR-329-3p and miR-518c in the functional expression of FOXM1, AXL, and eEF2K, we transfected LN229 and U87 GBM cells with control miR, miR-449b-5p, miR-329-3p or miR-518c and performed Western blot analyses. The results indicated that miR-449b-5p, miR-329-3p, and miR-518c treatments suppress FOXM1, AXL, and eEF2K expression, supporting the microRNA target prediction analyses and suggesting that miR-449b-5p, miR-329-3p, and miR-518c can simultaneously target eEF2K, AXL, and FOXM1 (Figure 2D,E).

### 3.4. miR-449b-5p, miR-329-3p, and miR-518c Inhibit the Proliferation of GBM Cells

**Figure 3:**
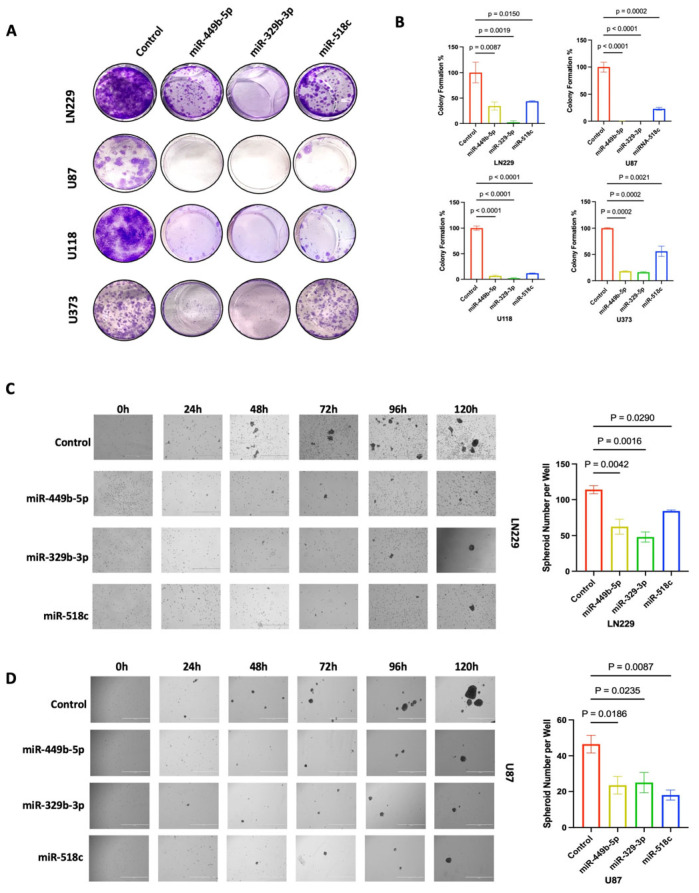
miR-449b-5p, miR-329-3p and miR-518c suppress colony formation and spheroid formation. (**A**,**B**) Analysis of total area of colony formation of LN229, U87, U118 and U373 cells treated with miR-449b-5p, miR-329-3p and miR-518c or control miR. miR-449b-5p, miR-329-3p and miR-518c significantly suppressed colony formation area of LN229 (*p* = 0.0087, *p* = 0.0019, *p* = 0.0150), U87 (*p* < 0.0001, *p* < 0.0001, *p* = 0.0002), U118 cells (*p* < 0.0001, *p* < 0.0001, *p* < 0.0001) and U373 cells (*p* = 0.0002, *p* = 0.0002, *p* = 0.0021). (**C**,**D**) Analysis of spheroid formation ability of LN229 and U87 cells in ultra-low attachment plates. The cells were monitored for five days. miR-449b-5p, miR-329-3p and miR-518c significantly suppressed spheroid formation of LN229 (*p* = 0.0042, *p* = 0.0016, *p* = 0.0290) and U87 cells (*p* = 0.0087, *p* = 0.0235, *p* = 0.0087). Original western blots are presented in Figure S3.

To determine the effect of miR-449b-5p, miR-329-3p, and miR-518c treatment on cell survival and clonogenic formation ability, we performed a clonogenic assay. For this assay, we transfected four different GBM cell lines (LN229, U87, U373, and U118) with control miR, miR-449b-5p, miR-329-3p, or miR-518c and found that the miR treatments significantly inhibited the colony formation ability of all GBM cell lines (Figure 3A,B). Among the three different miR treatments, miR-329-3p demonstrated the most consistent efficacy across all cell lines (Figure 3; LN229 *p* = 0.0019, U87 *p* < 0.0001, U118 *p* < 0.0068 and U373 *p* = 0.0118) (Supplementary Figure S2A; LN229 *p* = 0.0018, U87 *p* < 0.0004, U118 *p* < 0.0001 and U373 *p* = 0.0002) on total colony area and colony numbers. miR-449b-5p showed the second most effective suppression on colony formation area (Figure 3; LN229 *p* = 0.0087, U87 *p* < 0.0001, U118 *p* < 0.0001 and U373 *p* = 0.0002), while miR-518c treatment led to lesser inhibition than miR-449b-5p and miR-329-3p did (Figure 3; LN229 *p* = 0.0150, U87 *p* < 0.0002, U118 *p* < 0.0001 and U373 *p* = 0.0021).

### 3.5. miR-449b-5p, miR-329-3p, and miR-518c Treatment Inhibits GBM Spheroid Formation

Cancer stem cells (CSCs) are able to survive and form spheroids in low attachment plates or anchorage-independent cell culture conditions [33,34]. GBM is known for its stem cell-rich nature, leading to the failure of therapies including TMZ–chemotherapy [12,35]. To determine the effect of miR treatment on spheroid formation ability, we transfected LN229 and U87 cells with miR-449b-5p, miR-329-3p, and miR-518c and seeded the cells in ultra-low attachment plates for 5 days of monitoring. In both cell lines, namely, LN229 and U87, miR-449b-5p, miR-329-3p, and miR-518c treatments significantly suppressed the spheroid numbers, suggesting their potential in suppressing GBM stem cell proliferation and survival (Figure 3C,D; LN229: *p* = 0.0042, *p* = 0.0016, *p* = 0.0290, U87: *p* = 0.0186, *p* = 0.0235, *p* = 0.0087). Additionally, we performed Western blot analysis to assess the expression regulation of stemness markers including TWIST1, CD44 and CD133 (Supplementary Figure S2C) These markers exhibited differential levels of inhibition, suggesting heterogeneity in the regulatory mechanisms underlying the stem-like properties in these cells.

### 3.6. miR-449b-5p, miR-329-3p, and miR-518c Treatments Inhibit the Cell Migration and Invasion of GBM Cells

**Figure 4:**
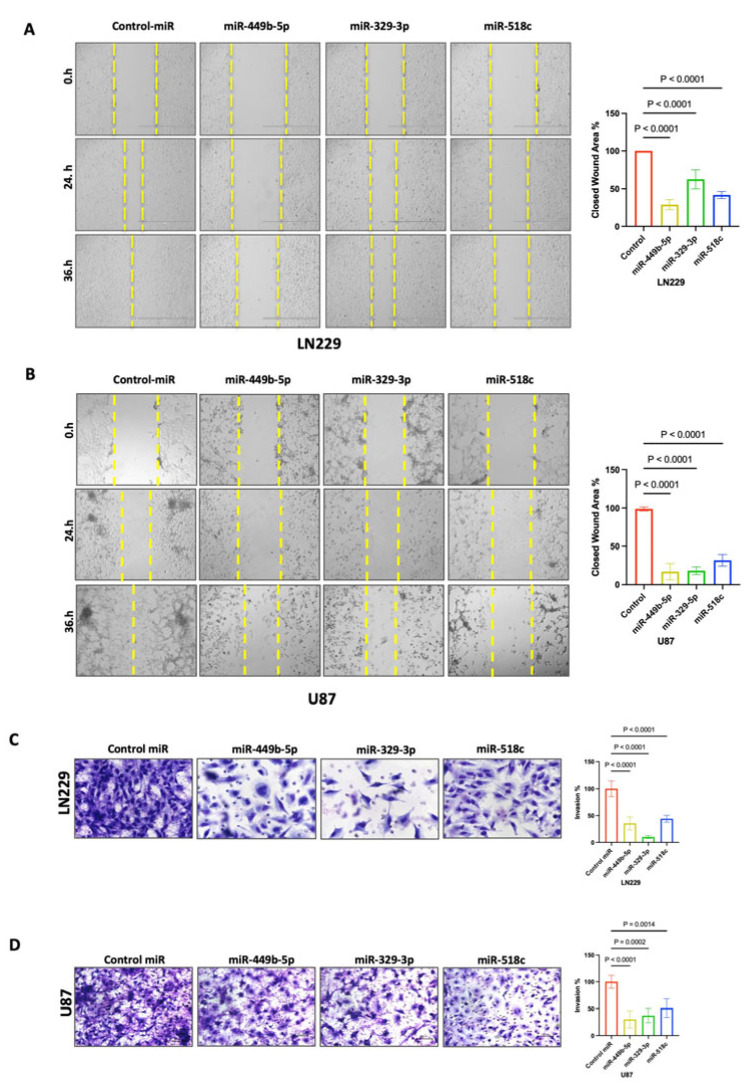
The suppressive effect of miR-449b-5p, miR-329-3p and miR-518c on cell migration and invasion. (**A**,**B**) LN229 and U87 cells transfected with 100 nM miR-449b-5p, miR-329-3p or miR-518c miRNA for 72 h were subjected to wound healing and in vitro transwell invasion assays. miR-449b-5p, miR-329-3p and miR-518c significantly suppressed the migration ability of both LN229 and U87 cells (*p* < 0.0001, *p* < 0.0001, *p* < 0.0001). (**C**,**D**) miR-449b-5p, miR-329-3p and miR-518c significantly suppressed the invasion ability of both LN229 (*p* < 0.0001, *p* < 0.0001, *p* < 0.0001) and U87 cells (*p* < 0.0001, *p* = 0.0002, *p* = 0.0014).

Due to their aggressive and invasive nature, GBM tumor tissue often infiltrates healthy brain tissue and cannot be entirely removed through surgery [2]. To assess the impact of miR-449b-5p, miR-329-3p, and miR-518c on the inhibition of invasiveness and motility capability of GBM cells, we performed in vitro migration (or wound healing) and Matrigel invasion assays. We treated LN229 and U87 cells with miR-449b-5p, miR-329-3p, or miR-518c and found that miR-449b-5p, miR-329-3p, and miR-518c suppress cell migration (Figure 4A,B; LN229, U87 all treatments *p* < 0.0001) and invasion ability of GBM cells (Figure 4C; LN229: *p* < 0.0001 and Figure 4D; U87: *p* < 0.0001, *p* = 0.0002, *p* < 0.0014).

### 3.7. miR-449b-5p, miR-329-3p, and miR-518c Treatments Promote Apoptotic Cell Death and Ferroptosis in GBM Cells

**Figure 5:**
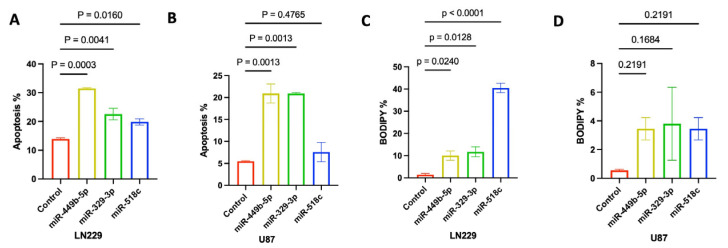
miR-449b-5p, miR-329-3p and miR-518c induce cell death mechanisms, apoptosis and ferroptosis. (**A**,**B**) Analysis of apoptosis by Annexin V/PI staining and flow cytometry of LN229 and U87 GBM cells after control miR, miR-449b-5p, miR-329,3p or miR-518c treatment. miR-449b-5p and miR-329-3p induced apoptotic cell death in LN229 cells (*p* = 0.0003, *p* = 0.0041) and U87 cells (*p* = 0.0013, *p* = 0.0013), while miR-518c only induced significant apoptosis in LN229 cells (*p* = 0.0160). (**C**,**D**) Analysis of ferroptosis by BODIPY staining and flow cytometry of LN229 and U87 GBM cells after control miRNA, miR-449b-5p, miR-329-3p or miR-518c treatment. miR-449b-5p, miR-329-3p and miR-518c induced significant BODIPY staining in LN229 cells (*p* = 0.0240, *p* = 0.0128, *p* < 0.0001).

To investigate the mechanism by which miR-449b-5p, miR-329-3p, and miR-518c exert their inhibitory effects on cell death mechanisms, we evaluated LN229 and U87 cells that were transfected with control miR, miR-449b-5p, miR-329-3p, or miR-518c with annexin V/propidium-iodide and BODIPY/DAPI staining to assess apoptosis and ferroptosis. miR-449b-5p, miR-329-3p, and miR-518c treatments significantly induced apoptosis in LN229 cells (Figure 5A; *p* = 0.0003, *p* = 0.0041 and *p* = 0.0161), and miR-449b-5p and miR-329-3p significantly induced apoptosis (Figure 5B; *p* = 0.0013, 0.0013) in U87 cells. While ferroptosis was not the predominant cell death mechanism in U87 cells, miR-518c induced stronger ferroptosis in LN229 cells compared to miR-449b-5p and miR-329-3p treatments (Figure 5C,D).

### 3.8. miR-449b-5p, miR-329-3p, and miR-518c Treatments Synergistically Enhance the Effects of TMZ

**Figure 6:**
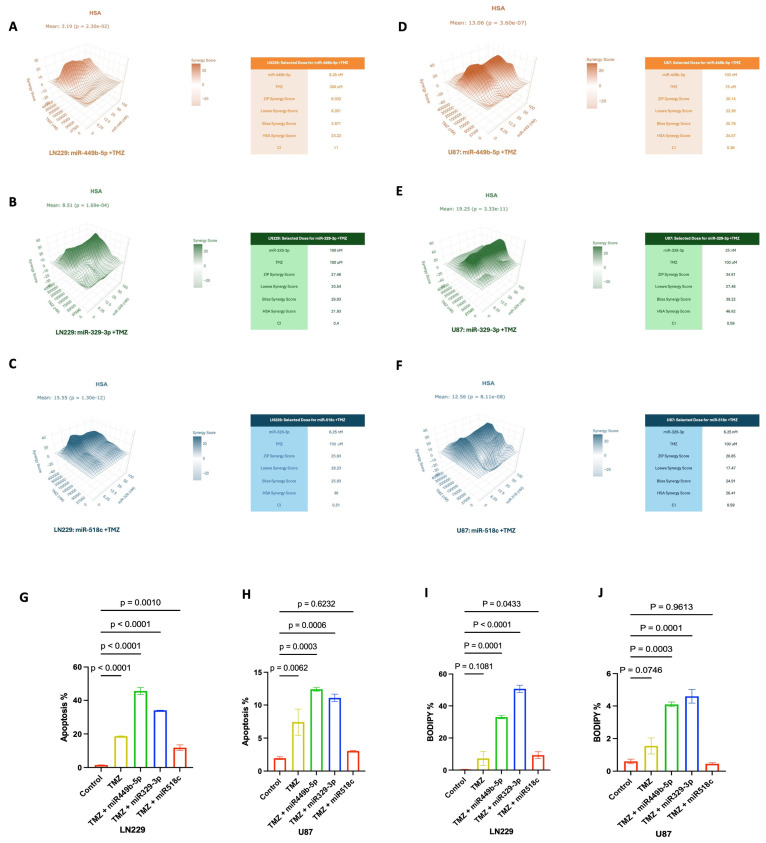
Combination of miR-449b-5p, miR-329-3p and miR-518c with Temozolomide (TMZ) leads to a synergistic inhibitory effect on GBM cell viability by enhancing TMZ. miR-449b-5p, miR-329-3p or miR-518c plus TMZ treatment enhanced the inhibitory effect of TMZ on cell viability of LN229 (**A**–**C**) and U87 cells (**D**–**F**). Most potent synergistic doses are represented in tables. (**G**,**H**) Analysis of LN229 and U87 cells after combinatory treatment with miR-449b-5p, miR-329-3p and miR-518c and TMZ. miR-449b-5p and miR-329-3p induced apoptotic cell death in both cell lines (LN229: *p* < 0.0001, *p* < 0.0001; U87: *p* = 0.0003, *p* = 0.0006), while miR-518c’s effect was only significant in LN229 cells (*p* = 0.0010). (**I**,**J**) Lipid peroxidation analysis of combinatory treatment of miR-449b-5p, miR-329,3p or miR-518c with TMZ. miR-449b-5p and miR-329-3p induced significant BODIPY signaling and enhanced the effect of TMZ treatment in both cell lines.

TMZ is the standard first-line chemotherapeutic agent used in the treatment of GBM; however, about 50% of GBM patients do not respond to TMZ, and those who initially respond quickly develop resistance, thereby limiting the effects of TMZ therapy [6]. To overcome these challenges and enhance the efficacy of TMZ, we treated GBM cells with miR-449b-5p, miR-329-3p and miR-518c and combined the miRs with TMZ to evaluate the combined inhibitory effects using SynergyFinder+, a computational tool for analyzing combinatorial drug effects and interactions. Overall synergy scores between 0 and 10 and CI values >1 were accepted as an additive effect, while scores above 10 and CI values <1 were accepted as an indicator of synergy. Four different synergy calculation methods were used for understanding the enhancement effect of combinatory treatment including Zip, Loewe, Bliss and HSA. We observed high synergistic scores for the overall synergy score of miR-518c+TMZ treatment. Along with that, combinatory treatment of miR-329-3p with TMZ at specific doses showed high synergistic scores in LN229 cells, while an additive effect was observed at other doses (Figure 6A–C). miR-449b-5p showed mostly additive effects (CI > 1), and the best dose combination observed at a dose of 6.25 nM combined with the 200 uM TMZ in LN229 cells (Figure 6A). miR-329-3p was found to be highly synergistic in the combination of 100 nM miR and 100 uM TMZ, with a synergy score above 20 (Figure 6B). In U87 cells, all miRNA and TMZ combination treatments led to highly synergistic effects, as indicated by scores above 20 (Figure 6D–F). Specifically, 25 nM miR-329-3p treatment in combination with 100 uM TMZ showed the highest synergistic score, which was found to be up to 46.62 in U87 cells (Figure 6E).

### 3.9. Combination of miR-449b-5 or miR-329-3p with TMZ Enhances Apoptosis and Ferroptosis

To investigate the effect of combinational therapy involving control miR, miR-449b-5p, miR-329-3p, or miR-518c and temozolomide (TMZ) on GBM cell death, LN229 and U87 cells were transfected with control miRNA, miR-449b-5p, miR-329-3p, or miR-518c. Forty-eight hours post-transfection, the cells were treated with TMZ at its IC_50_ concentrations, which was assed earlier (Supplementary Figure S2B). miR-449b-5p and miR-329-3p enhanced apoptotic cell death in both cell lines when combined with TMZ (Figure 6G–J). Interestingly, miR-518c showed a weaker impact compared to other miRNAs. Furthermore, miR-449b-5p and miR-329-3p enhanced TMZ-induced ferroptosis in both cell lines, whereas miR-518c had no significant impact on ferroptosis in U87 cells.

### 3.10. In Vivo Treatment with miR-329-3p Suppresses the Growth of GBM Tumor Xenografts in Mice, Inhibits FOXM1-eEF2K/AXL, and Induces Apoptosis

**Figure 7:**
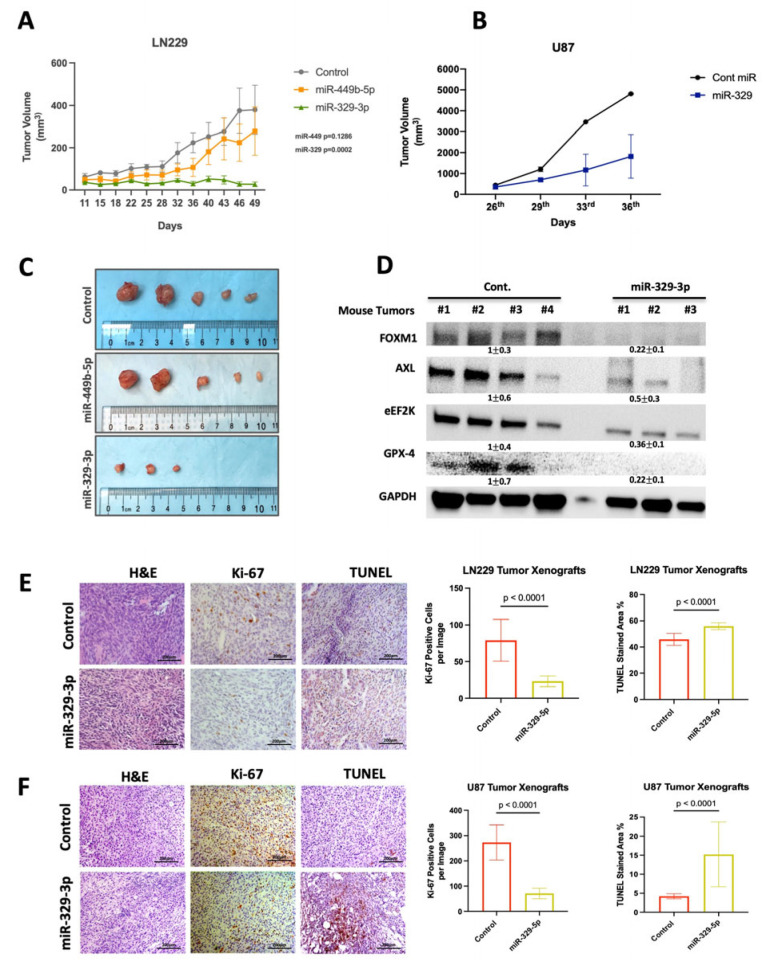
In vivo systemic miR-329-3p nanotherapy suppresses growth of GBM tumor xenografts in a flank model by inhibiting FOXM1, AXL and eEF2K and inducing apoptotic cell death. (**A**,**C**) An in vivo LN229 GBM flank model was used for assessing the in vivo therapeutic efficacy of miR-449b-5p and miR-329-3p on in vivo tumor growth in mice. Tumor sizes were traced for 49 days. After the 36th day, while miR-449b-5p exhibited insignificant effects on tumor growth, miR-329-3p showed significant tumor-suppressive effects. Two out of five tumors that had been treated with miR-329-3p regressed. (**B**) miR-329-3p administration suppressed tumor growth in an in vivo U87 GBM flank model. (**D**) At the end of treatments, LN229 tumors were removed from mice and analyzed by Western blotting for evaluation of target downregulation. miR-329-3p suppressed FOXM1, AXL and eEF2K as well as GPX-4, a marker for assessing ferroptosis. (**E**,**F**) Immunohistochemistry analysis revealed that miR-329-3p treatment suppresses intra-tumoral cell proliferation as indicated by reduced (Ki-67) staining and induction of apoptosis (TUNEL) (*p* < 0.0001, *p* < 0.0001). Original western blots are presented in Figure S3.

Based on in vitro screening, miR-449b-5p and miR-329-3p were identified as the most potent candidates for use in in vivo targeting of GBM murine models. Treatment with miR-329-3p significantly suppressed tumor growth in the LN229 tumor xenograft model in mice with no observed toxicity (Figure 7A). Notably, complete tumor regression was observed in two out of five of the LN229 tumor model mice following miR-329-3p administration (Figure 7C). In contrast, the notable difference observed between the miR-449b-5p treatment group and the control group on day 36 was no longer significant by day 49 (Figure 7A). Western blot analysis revealed that the in vivo administration of miR-329-5p nanoparticles suppressed the expression of FOXM1, AXL, and eEF2K along with GPX4 (Figure 7D), the central regulator that protects cells against ferroptosis. Moreover, to validate the tumor suppressive effect of miR-329-3p, we used a U87 GBM flank model. Single lipid nanoparticles (SLNPs) conjugated with miR-329-3p (0.15 mg/kg), administered systemically via intraperitoneal (i.p.) injection, successfully suppressed tumor growth in the U87 in vivo model (Figure 7B). In addition, immunohistochemical analysis of control miR- and miR-329-5p-treated LN229 and U87 tumor xenografts revealed significantly increased TUNEL staining and reduced Ki-67 expression, indicating that miR-329-5p treatment promotes apoptosis and suppresses cell proliferation, respectively, in the GBM in vivo flank model (Figure 7E,F).

## 4. Discussion

Glioblastoma multiforme (GBM) is an incurable primary brain tumor that has a 13-month median survival with the currently available therapies. Therapy failures are attributed to its highly invasive nature, molecular heterogeneity, the presence of a significant number of CSCs, therapy resistance and the lack of effective targeted therapies [2,9,36,37,38]. Radiotherapy and chemotherapy (i.e., TMZ) often fail to prolong patient survival, necessitating the identification of novel therapeutic targets and treatment strategies for GBM patients.

MicroRNAs, a subclass of small non-coding RNAs, regulate post-transcriptional gene expression and offer multitargeting potential in cancer by binding to multiple mRNA targets and modulating various signaling pathways involved in tumor biology [14,39]. Several studies have shown that miRNAs play key roles in GBM tumorigenesis [40]. While miR-449b-5p, miR-329-3p, and miR-518c have been reported to be downregulated and play a regulatory role in various cancers such as cervical cancer, lung cancer, osteosarcoma, triple-negative breast cancer, gastric, and colorectal cancer, their downstream targets’ mechanisms of action were not well understood [41,42,43,44,45,46,47,48,49,50]. In this study, after extensive analyses using various miRNA target prediction algorithm databases, we identified miR-449b-5p, miR-329-3p, and miR-518c as potential important oncogenic regulators of clinically significant oncogenic molecules such as eEF2K, AXL, and FOXM1 for potential therapeutic intervention in GBM. We have recently demonstrated that FOXM1, AXL, and eEF2K are potential oncogenic targets for promoting GBM cell proliferation, migration and invasion [25]. In the current study, we demonstrated that these oncogenic molecules are highly upregulated in GBM patient tumors in comparison to other brain tumors and normal tissues, suggesting that they are clinically significant oncogenic drivers of GBM. Therefore, in addition to revealing the role of miR-449b-5p, miR-329-3p, and miR-518c, our study provides, to the best of our knowledge, the first evidence using these tumor suppressor microRNAs as a potential therapeutic tool in GBM tumor models to potentially target eEF2K, AXL, and FOXM1.

Furthermore, our results indicate that miR-449b-5p, miR-329-3p, and miR-518c act as tumor suppressor miRs and that their expression suppresses GBM cell proliferation, migration, and invasion and stemness while simultaneously inducing apoptosis and ferroptosis; these findings highlight the therapeutic potential of miR treatments in GBM patients through their multitargeting potential. More importantly, our in vivo results demonstrated that miR-329-5p nanotherapy significantly suppresses GBM tumor growth by inhibiting FOXM1, AXL, and eEF2K expression levels and promoting apoptosis, suggesting that this innovative approach may be a potential novel therapy for GBM. Furthermore, the combination of miR-329-5p with TMZ leads to a synergistic effect in GBM cells by enhancing TMZ’s cytotoxicity, through their ability to downregulate key genes such as FOXM1, AXL, and eEF2K, which are involved in cell proliferation, survival, invasion and tumorigenic pathways. This combined approach may overcome TMZ resistance, reduce tumor growth, and improve therapeutic outcomes in GBM treatment. Therefore, future studies should evaluate the combined effect of miR-329-5p and TMZ in in vivo preclinical models of GBM.

eEF2K is classified as an oncogenic alpha kinase and has been identified as being upregulated in GBM [19]. Previously, eEF2K was associated with autophagy through Akt signaling in gliomas [51,52]. Studies have also associated eEF2K with promoting glioblastoma progression, particularly through its involvement in processes such as migration, invasion, and apoptosis, especially when combined with temozolomide [53,54]. FOXM1, a transcription factor that is frequently upregulated in GBM, contributes to tumor progression by regulating genes involved in proliferation, angiogenesis, epithelial–mesenchymal transition, invasiveness, and stemness, making it a promising target for therapeutic intervention [55,56,57]. Similarly, AXL has emerged as a potential therapeutic target due to its role in supporting tumor growth, invasion, and motility in a range of malignancies through PI3K/AKT/mTOR signaling, JAK/STAT signaling, and the NF-κB and RAS/MAPK pathways [20,58]. In GBM, elevated AXL expression has been linked to aggressive tumor behavior, while its inhibition has been shown to reduce invasion and migration while triggering apoptosis in preclinical models [59,60,61,62]. We have previously demonstrated that eEF2K, AXL, and FOXM1 are upregulated in GBM patients’ tumor samples and exhibit variable expression levels across different GBM cell lines, promoting cell proliferation, stemness and motility through their interactions with each other [25]. Their interactions with each other and the suppressive role of their inhibition in GBM tumorigenesis suggest their potential as candidates for targeted therapies [24].

miR-329 is downregulated in GBM, and its inhibition promotes cell proliferation, while its overexpression impairs cell proliferation and 3D colony formation [63,64]. Our results also confirmed the effects of miR-329-3p suppression on in vitro cell growth and colony formation. Furthermore, we observed significant inhibition in four different GBM cell lines with different genetic features and a marked inhibitory effect on cell proliferation compared to the other candidate miRs. In addition, we demonstrated that miR-329-3p is the best suppressor of eEF2K expression and an important player both in in vitro GBM cell proliferation and invasion and in in vivo tumorigenesis of GBM. miR-329-3p significantly suppressed spheroid formation, indicating its role in GBM stemness, drug resistance and invasiveness and its potential role as a tumor suppressor. As expected, miR-329-3p treatment consistently induced programmed cell death pathways such as apoptosis and ferroptosis, especially in combination with TMZ in GBM cells. miR-329-3p demonstrated strong synergistic effects with TMZ, even at low concentrations and TMZ doses below its IC_50_. Most importantly, the in vivo LN229 and U87 GBM xenograft flank models confirmed the potency of miR-329-3p, demonstrating consistent tumor growth suppression and regression. Yet, it needs to be acknowledged that a limitation of our study is the use of a subcutaneous flank xenograft model rather than an orthotopic intracranial GBM model, which more closely recapitulates the blood–brain barrier-associated therapeutic constraints. In alignment with the in vitro findings, miR-329-3p in vivo nanotherapy suppressed FOXM1, AXL, eEF2K and Ki-67 expression and induced TUNEL positivity, underscoring miR-329-3p as a strong multitargeting strategy for GBM tumorigenesis.

Several studies showed that miR-449b-5p is downregulated in various cancers, including osteosarcoma, ovarian cancer, and GBM patients [41,42,65]. We found that miR-449b-5p acts as a tumor suppressor miRNA and suppresses cell proliferation, cell migration–invasion, and spheroid formation. This finding is consistent with a recent study that showed that the upregulation of miR-449b-5p suppresses cell proliferation and spheroid formation [41]. In our study, we also investigated the regulatory effect of miR-449b-5p on the apoptotic cell death mechanism and ferroptosis in GBM and demonstrated that it induces apoptosis and ferroptosis in combination with TMZ and that it enhances the effect of TMZ on apoptotic cell death and ferroptosis. These results are consistent with our previous study, in which we demonstrated that miR-449b and miR-329 are potential players in GBM 3D cell growth and effective enhancers of TMZ [66]. In contrast to the in vitro success of miR-449b-5p, we found that miR-449b-5p had insignificant effects on in vivo xenograft LN229 tumor model tumor growth. We would like to highlight that although miR-449b-5p was found to be effective in in vitro studies, its lack of significant in vivo efficacy may be attributed to the suboptimal dosing.

Our study indicated that miR-518c is predicted to bind to eEF2K, AXL and FOXM1 mRNA in the 3’-UTR region and suppressed their protein expression. Previously, miR-518c was shown to suppress cell proliferation and induce apoptotic markers, such as cleaved caspase-3 and TUNEL positivity [67]. We demonstrated that miR-518c significantly suppressed cell proliferation, migration, invasion, and spheroid formation and induced both apoptotic cell death and ferroptosis in LN229 cells, but not in U87 cells, indicating a cell line–specific effect. miR-518c also exhibited strong synergistic effects with TMZ in reducing cell proliferation; however, its ability to induce cell death was diminished when used in combination with TMZ. Due to its relatively low overall potency in vitro and lack of consistent efficacy in combination with TMZ, miR-518c was not selected for in vivo experiments. Future studies may explore other cell death mechanisms to better understand its role alone or when combined with TMZ.

## 5. Conclusions

In summary, our findings are, to the best of our knowledge, the first to show that miR-449b-5p, miR-329-3p, and miR-518c act as powerful multitargeting regulators of GBM growth and therapy resistance through the regulation of the FOXM1/AXL-eEF2K axis. They suppress key malignant behaviors, including proliferation, invasion, spheroid formation, and survival, and when combined with TMZ, they further enhance cell death through apoptosis and ferroptosis through combined FOXM1, AXL, and eEF2K inhibition. Notably, miR-329-3p demonstrated strong antitumor activity in vivo, underscoring its potential as a promising therapeutic lead.

While our study is limited by reliance on Western blot analysis to assess target protein suppression and the usage of a flank tumor model, the results provide a compelling rationale for targeting FOXM1, eEF2K and AXL and advancing these miRNAs, especially miR-329-3p, into orthotopic GBM systems and refining delivery strategies capable of crossing the blood–brain barrier.

Together, this work supports the development of miRNA-based therapeutics as a practical, multitargeted approach to treat and control the growth, invasion and progression of highly heterogenous GBM tumors and to overcome drug resistance.

In future studies, downstream pathway regulation of miRs through eEF2K, AXL and FOXM1 signal suppression would provide a more comprehensive understanding of GBM molecular mechanisms. Moreover, development of in vivo orthotopic xenograft models and novel nanocarriers to facilitate the transportation of miR-329-3p through the blood–brain barrier is a crucial step for the translation of miR-based therapies into the clinic.

## Data Availability

The datasets generated and/or analyzed in the current study are available from the corresponding author on reasonable request.

## Supplementary Materials

The following supporting information can be downloaded at: https://www.mdpi.com/article/10.3390/cancers18091479/s1, Figure S1: CCGA expression analysis of eEF2K, AXL and FOXM1; Figure S2: (A) Colony number analysis of LN229, U87, U373 and U118 cells after control miR, miR-449, miR-329 and miR-518 treatment. (B) Assessment of IC50 viability values of LN229 and U87 cells after TMZ treatment through MTS assay. (C) Western blot analysis of LN229 and U87 cells for stemness markers; Figure S3: Original western blots.

## Abbreviations

The following abbreviations are used in this manuscript:

GBM	Glioblastoma Multiforme
miRNA	microRNA
siRNA	Small Interfering RNA
eEF2K	Eukaryotic Elongation Factor 2 Kinase
FOXM1	Forkhead box transcription factor
TMZ	Temozolomide
