# Supplementary material for: Tumor-Suppressive microRNA Therapy Inhibits Growth of Glioblastoma Multiforme Xenografts
Source: Cancers (Basel). 2026 May 4;18(9):1479. doi: 10.3390/cancers18091479 (PMC13162599; doi:10.3390/cancers18091479)
Supplement: Supplementary file 1 [file cancers-18-01479-s001.zip › cancers-4222283-supplementary.pdf]

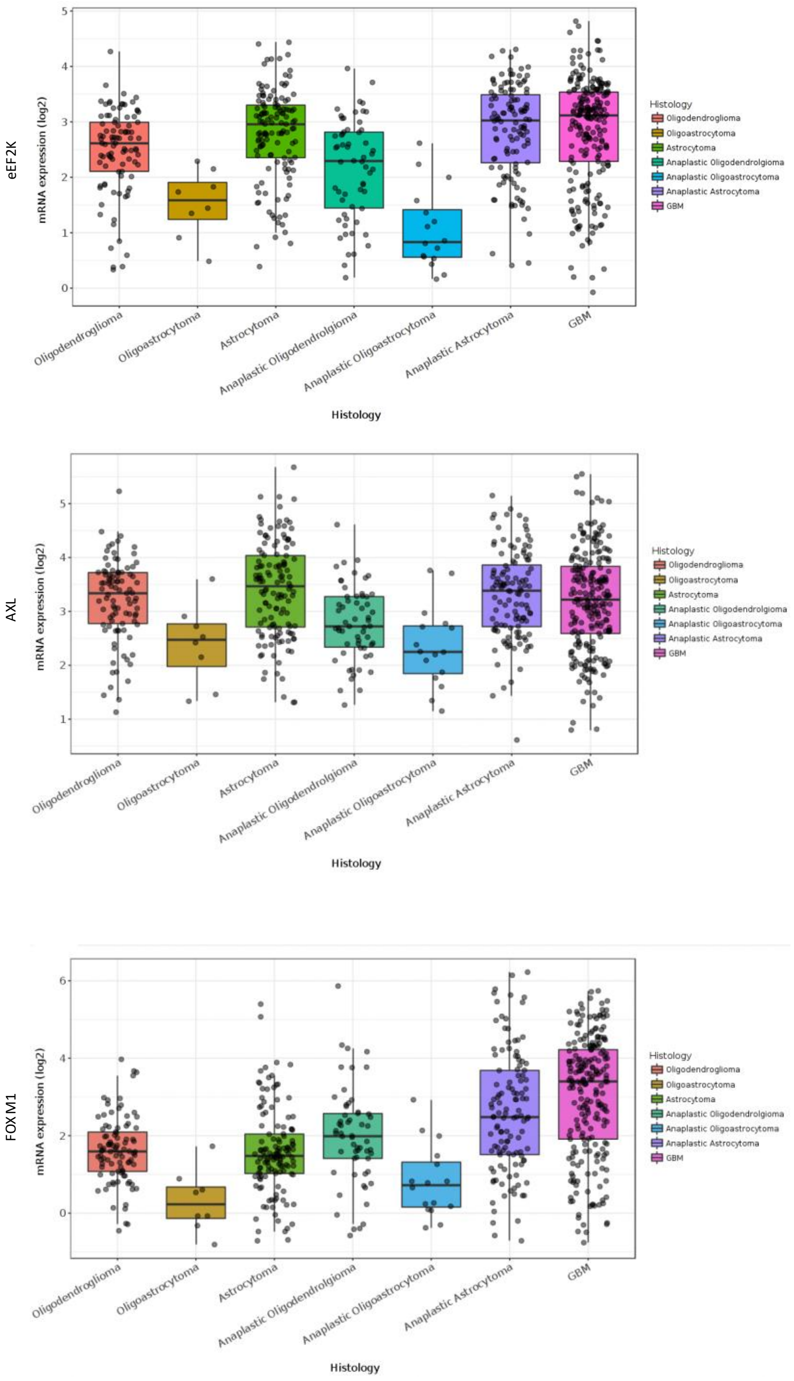

Sup. Fig. S1 *CCGA expression analysis of eEF2K, AXL and FOXM1*

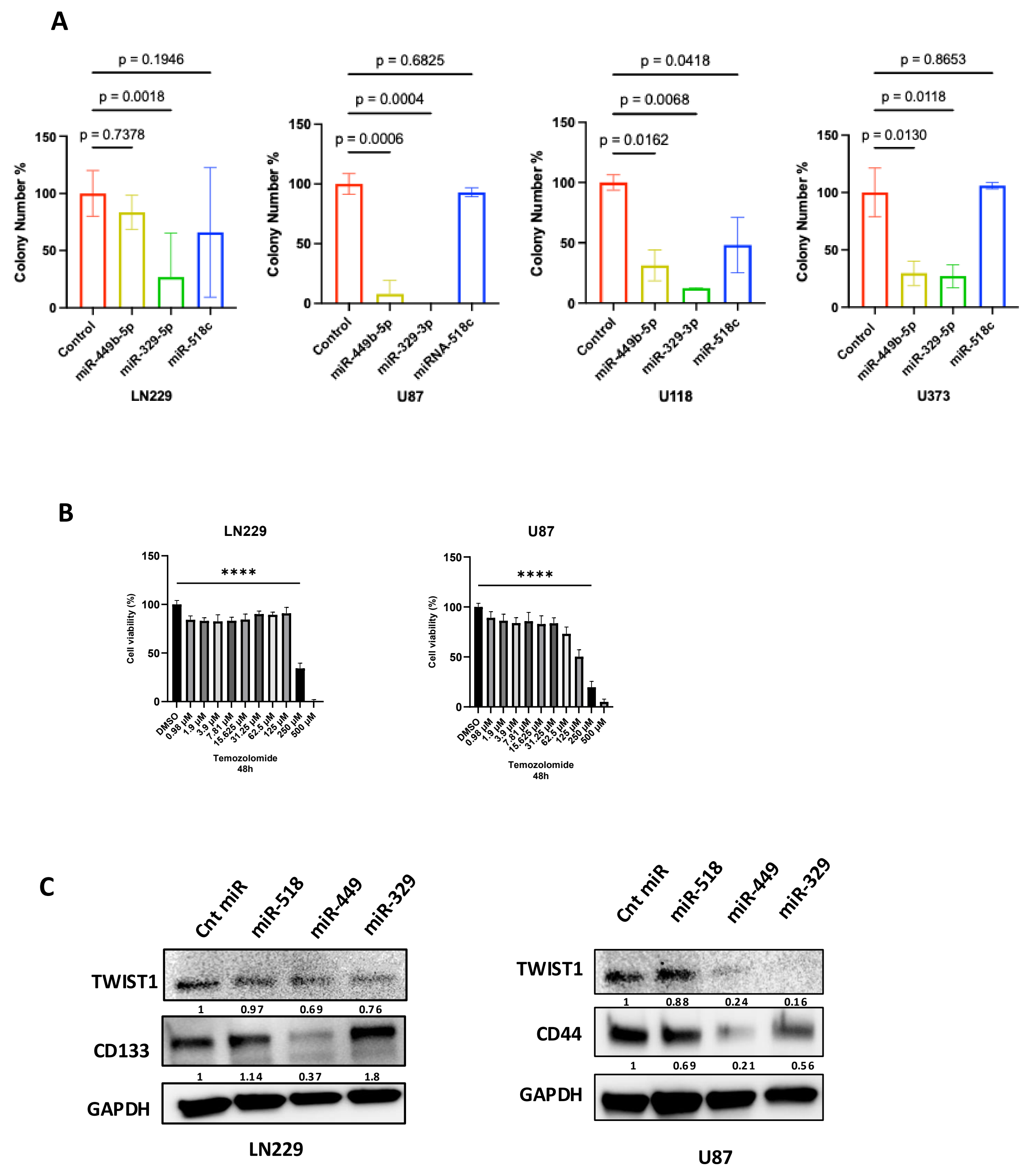

Sup. Fig. S2 *A) Colony Number Analysis of LN229, U87, U373 and U118 cells after control miR, miR-449, miR-329 and miR-518 treatment B)Assesment of IC50 viability values of LN229 and U87 cells after TMZ treatment through MTS assay C) Western blot anaylsis of LN229 and U87 cells for stemness markers.*
