# Supplementary figures and images for: Tumor-Suppressive microRNA Therapy Inhibits Growth of Glioblastoma Multiforme Xenografts
Source: Cancers (Basel). 2026 May 4;18(9):1479. doi: 10.3390/cancers18091479 (PMC13162599; doi:10.3390/cancers18091479)

# **WB Membranes**

Figure 1G

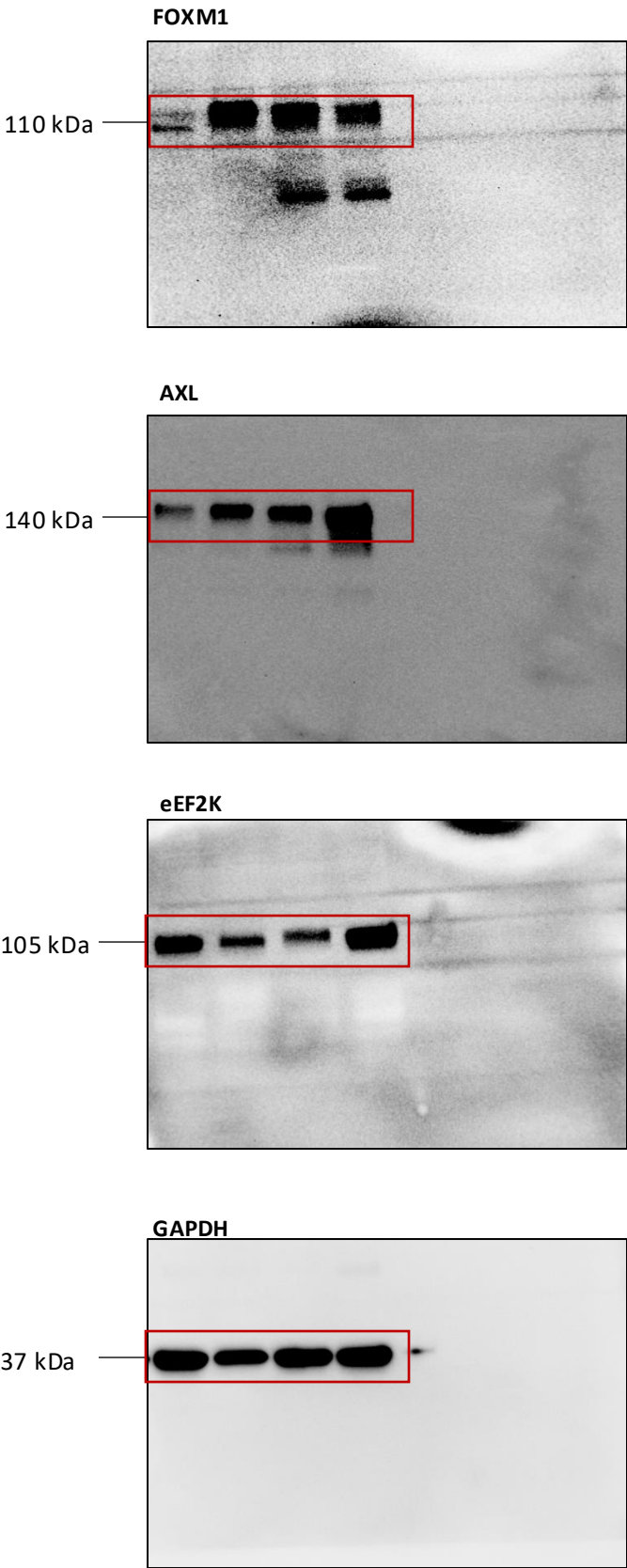

Figure 2D

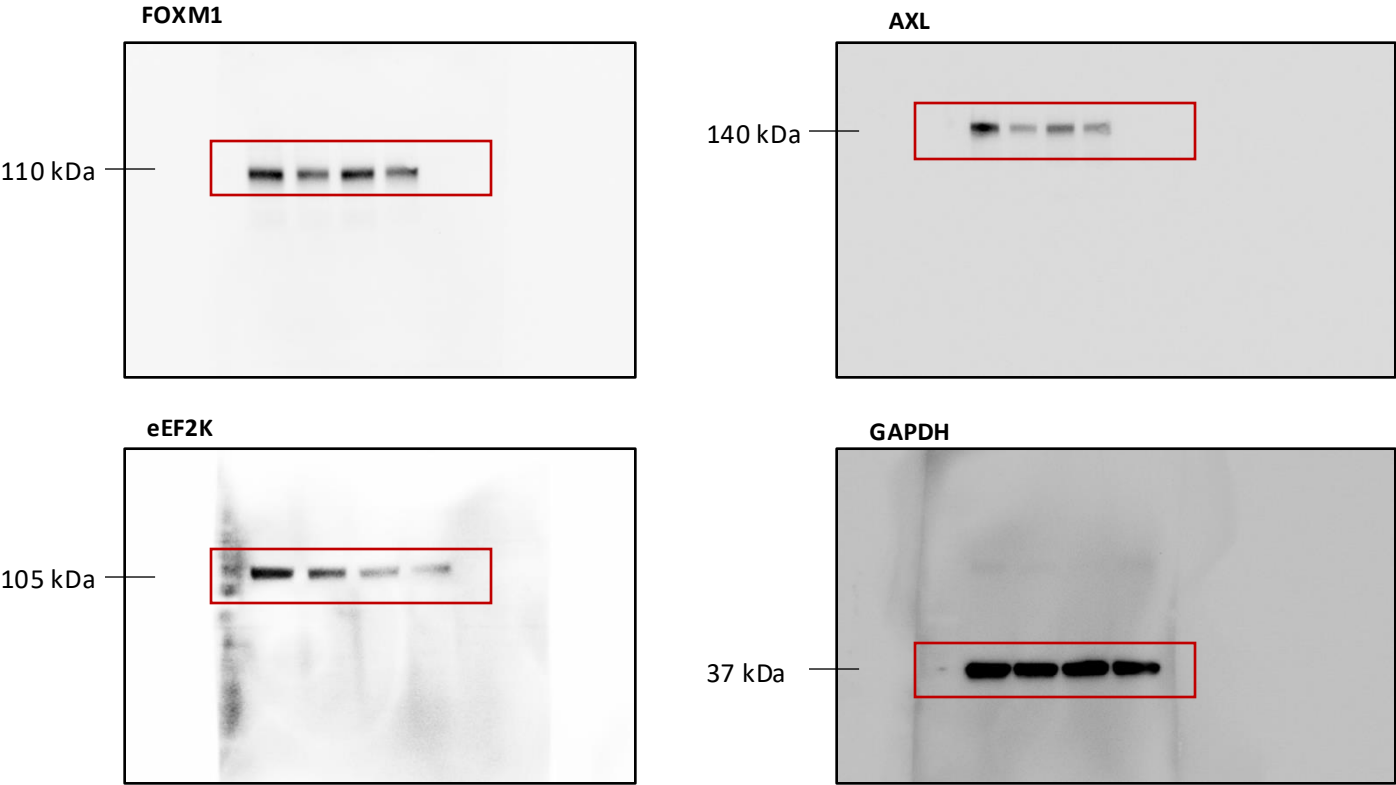

Figure 2E

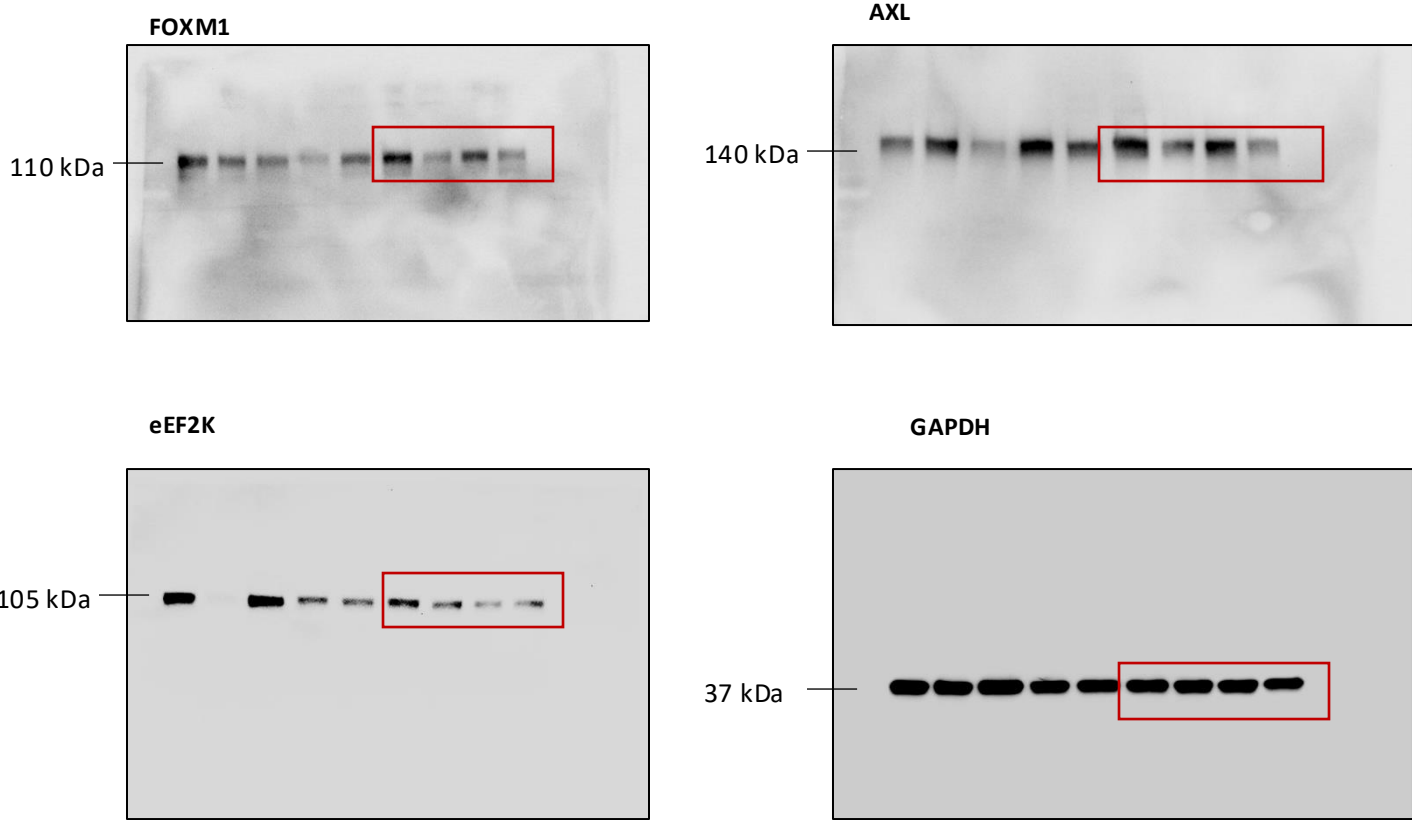

Figure 8D

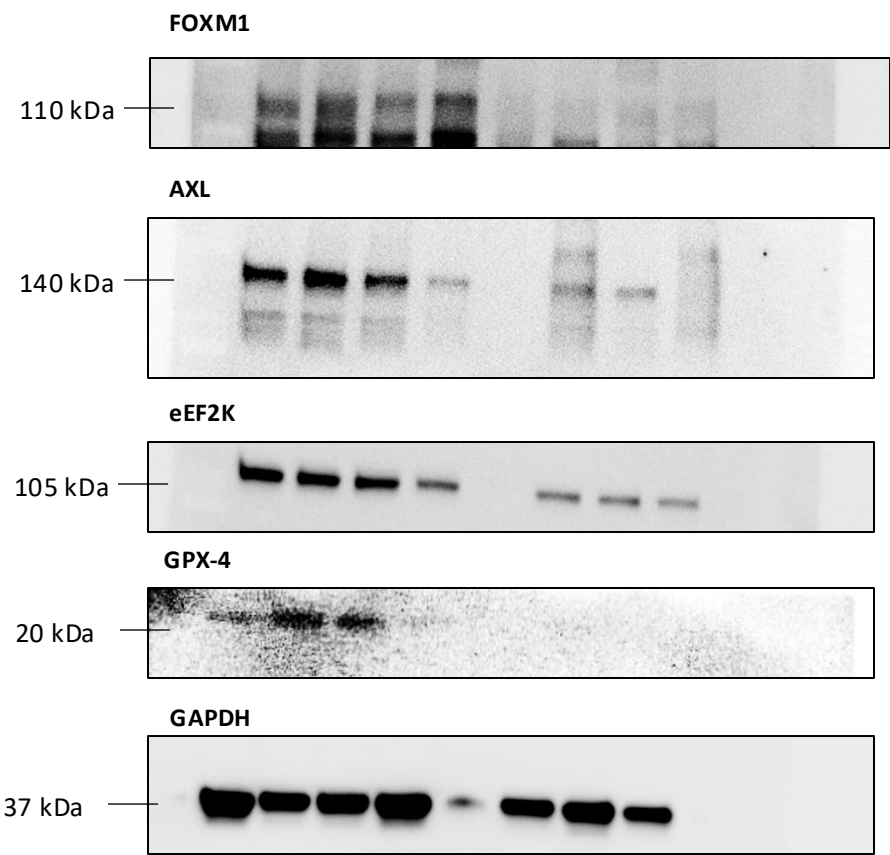

# Supplementary Figure 1C

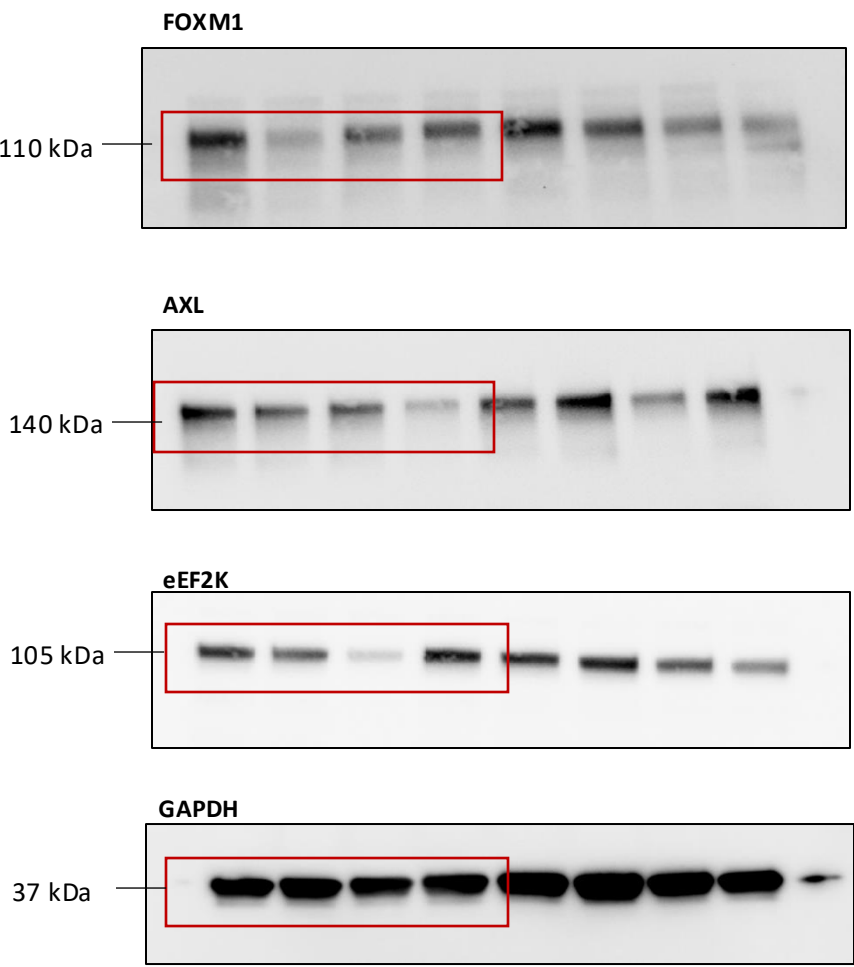

# Supplementary Figure 1D

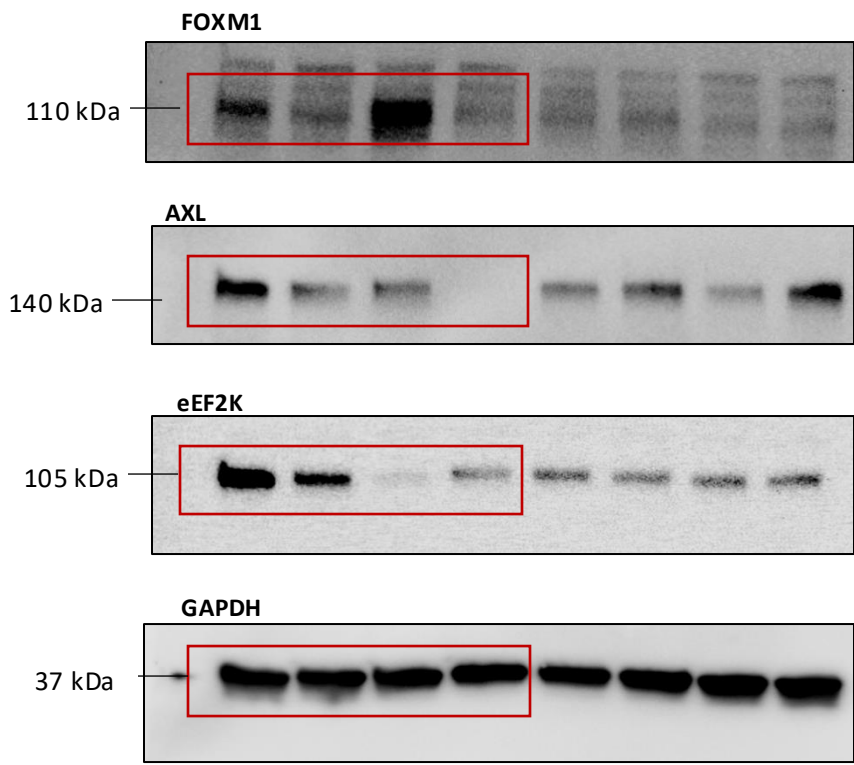

Supplement: Supplementary file 1 [file cancers-18-01479-s001.zip › Figure S3.pdf]
